# The impact of PICCO monitoring on traumatic shock: a systematic review and meta-analysis

**DOI:** 10.3389/fmed.2025.1578348

**Published:** 2025-11-14

**Authors:** Aihua Lin, Zhangyue Lin, Ke Xu, Jiali Chen, Xun Ni

**Affiliations:** 1 Department of Critical Care Medicine, The Affiliated Suqian Hospital of Xuzhou Medical University, Suqian, China; 2 Department of Critical Care Medicine, Suqian Hospital of Nanjing Drum Tower Hospital Group, Suqian, China; 3 Department of Clinical Medicine, Yangzhou University Medical College, Yangzhou, China

**Keywords:** pulse indicator continuous cardiac output, traumatic shock, hemorrhagic shock, meta-analysis, central venous pressure

## Abstract

**Purpose:**

This study aims to provide a systematic review and meta-analysis of Pulse Indicator Continuous Cardiac Output (PICCO) compared with conventional central venous pressure (CVP) monitoring in the treatment of traumatic shock.

**Methods:**

A systematic literature retrieval was conducted in databases including PubMed, Web of Science, Cochrane Library, Embase, and China National Knowledge Infrastructure (CNKI) from database inception to October 22, 2024. Keywords such as “PICCO,” “traumatic shock,” and “hemorrhagic shock” were used. Retrieved studies were screened according to pre-determined inclusion and exclusion criteria. The methodological quality and risk of bias were assessed using the Newcastle-Ottawa Scale (NOS) for cohort studies and the Cochrane “risk of bias” tool for randomized controlled trials (RCTs). Outcomes, including mortality, duration of mechanical ventilation, and length of ICU stay, were extracted and meta-analyzed.

**Results:**

A total of 15 studies comprising 1,188 patients were included, with 597 monitored by PICCO and 591 by routine CVP. The risk of bias was assessed as low for all studies. PICCO-monitored patients showed a significantly shorter duration of mechanical ventilation compared to the control group [SMD in random effects model: −1.66; 95% CI: (−2.38, −0.94)]. However, no significant differences were found in mortality or length of ICU stay.

**Conclusion:**

PICCO monitoring can improve the prognosis of traumatic shock patients by shortening the duration of mechanical ventilation, but it does not significantly affect mortality or length of ICU stay. Given the limitations of the included studies, further exploration is warranted to verify these conclusions.

## Introduction

Traumatic shock is a life-threatening subset of hypovolemic shock primarily caused by acute hemorrhage combined with major soft tissue injury, leading to a critical reduction in circulating blood volume and impaired tissue perfusion in intensive care unit (ICU) (1). Specifically, traumatic shock includes two closely related entities: traumatic hemorrhagic shock, which results from acute blood loss accompanied by extensive soft tissue damage and subsequent inflammatory and coagulopathic responses, and traumatic hypovolemic shock, characterized by significant fluid loss without active hemorrhage but with soft tissue injury and immune activation (2, 3). Severe trauma-hemorrhage and coagulation disorder are major cause of death. Effective management of traumatic shock relies heavily on accurate hemodynamic monitoring to guide fluid resuscitation and vasoactive therapy. The monitoring method commonly used in current is central venous pressure (CVP) monitoring, which is a traditional hemodynamic parameter that does not directly measure cardiac output (CO) and has recently been reported to have several limitations, including inefficiently response to hemodynamic changes and frequent fluctuations in measurements (4–6). Consequently, the development of innovative monitoring techniques is likely to enhance the therapeutic outcomes for patients with traumatic shock.

Pulse indicator continuous cardiac output (PICCO) system is a hemodynamic monitoring method technology that combines transpulmonary thermodilution (TPTD) for volumetric calibration with continuous arterial pulse contour analysis to assess cardiac function and fluid status (7). By using these techniques, PICCO allows for monitoring of numerous physiological variables including global end diastolic volume, intrathoracic blood volume (ITBV), and cardiac index (CI). With these measurements, PICCO can accurately reflect the hemodynamic status of a patient. Several studies have evaluated the clinical efficacy of PICCO. However, the conclusions in them exhibited inconsistent. One study compared treatment based on either PICCO-derived physiological values or CVP monitoring and found that PICCO was not able to reduce the 28-day mortality risk (8). In contrast, another study demonstrated that PICCO system can improve clinical outcomes in critically ill patients with acute respiratory distress syndrome (ARDS) (9). Taken together, systematic analysis of the clinical efficacy of PICCO on traumatic shock patients is still under-investigated.

This study aims to investigate the efficacy or futility of PICCO-based hemodynamic monitoring in patients with traumatic shock and hemorrhagic shock. While the PICCO system has been widely evaluated and shown to improve outcomes in septic shock patients, such as reducing mortality and shortening ICU stay and mechanical ventilation duration (10), evidence concerning its effectiveness in traumatic or hemorrhagic shock remains limited. By capitalizing on meta-analytic techniques, the current study examined the relationship between PICCO monitoring and clinical outcomes—namely, mortality, duration of mechanical ventilation, and length of ICU stay—in patients with traumatic or hemorrhagic shock, compared with conventional CVP monitoring. By analyzing the clinical efficacy of PICCO specifically in this population, our study provides new evidence and insights for fluid management in critical care settings where traumatic or hemorrhagic shock is prevalent.

## Materials and methods

Following the Cochrane Handbook and PRISMA guidelines, we prospectively registered our systematic review and meta-analysis protocol (registration number: CRD420251138853).

### Search strategy and eligible criteria

A literature search in various databases (Pubmed, Web of Science, Cochrane Library, Embase and China National Knowledge (CNKI)) was conducted on October 22nd, 2024 by using keywords such as “pulse index contour continuous cardiac output (PiCCO),” “traumatic shock,” and “hemorrhagic shock.” The detailed search strategy used for each database were listed in Supplementary Table S1.

Studies were included if they met the following criteria:

(1) The study was conducted on patients with traumatic shock, defined as a form of hypovolemic shock caused primarily by acute hemorrhage and major soft tissue injury, resulting in reduced circulating blood volume and impaired tissue perfusion (1).
(2) The study was a comparative one, in which the intervention group adopted PICCO, while the control group used conventional monitoring measures;
(3) The study contained indicators to assess the efficacy, including mean arterial pressure (MAP), CVP, blood lactate value, etc.;
(4) Full text was available.

Studies were excluded based on the following criteria:

(1) Review/meeting/case report;
(2) Animal/cellular studies;
(3) PICCO technology was not applied in the study;
(4) Studies with patient populations not limited to traumatic shock.

For studies reporting mortality as an outcome, we extracted the specific definition of mortality used in each study (e.g., 28-day mortality, ICU mortality, in-hospital mortality). In this meta-analysis, “28-day mortality” refers to deaths from any cause within 28 days after randomization or admission, while “ICU mortality” refers to deaths occurring during the index ICU stay.

### Data acquisition and quality assessment

For potentially eligible studies, two reviewers independently check the eligibility of full-text articles using standard forms after title and abstract screening and extracted the following data from each eligible study: mortality, duration of mechanical ventilation, and length of ICU stay. For cohort study, the methodological quality and risk of bias was evaluated by the Newcastle-Ottawa Scale (NOS) tool; for RCT study, the methodological quality and risk of bias was assessed by using the Cochrane “risk of bias” tool. Conflicts between the 2 reviewers were resolved by a third reviewer.

### Statistical analysis

Three outcomes were considered for meta-analysis: (a) mortality, (b) duration of mechanical ventilation, and (c) length of ICU stay. Meta-analysis was performed with the use of the meta package in Review Manager version 5.3. The results were illustrated as forest plots. *p* < 0.05 was considered as statistical significance. Risk ratio (RR) and 95% confidence interval (CI) were calculated to assess binary data, while standard mean difference (SMD) and 95% CI were calculated for continuous data. Statistical heterogeneity between the included studies was evaluated using the *I*^2^ and Tau^2^ statistic. Significant heterogeneity was defined as *p* < 0.05. *I*^2^ values of 25, 50, and 75% are considered low, moderate, and high estimates, respectively. Results were analyzed using a fixed-effect model when heterogeneity estimates were low, and a random-effects model was employed when heterogeneity estimates were moderate or high. Funnel plots were used to evaluate publication bias based on standard errors (SE) and corresponding measures. *p* < 0.05 was considered statistically significant. Sensitivity analysis was adopted by sequentially omitting each included study and estimating the overall impact of the study on the pooled results.

## Results

### Characteristics and quality assessment of the included studies

**Figure 1:**
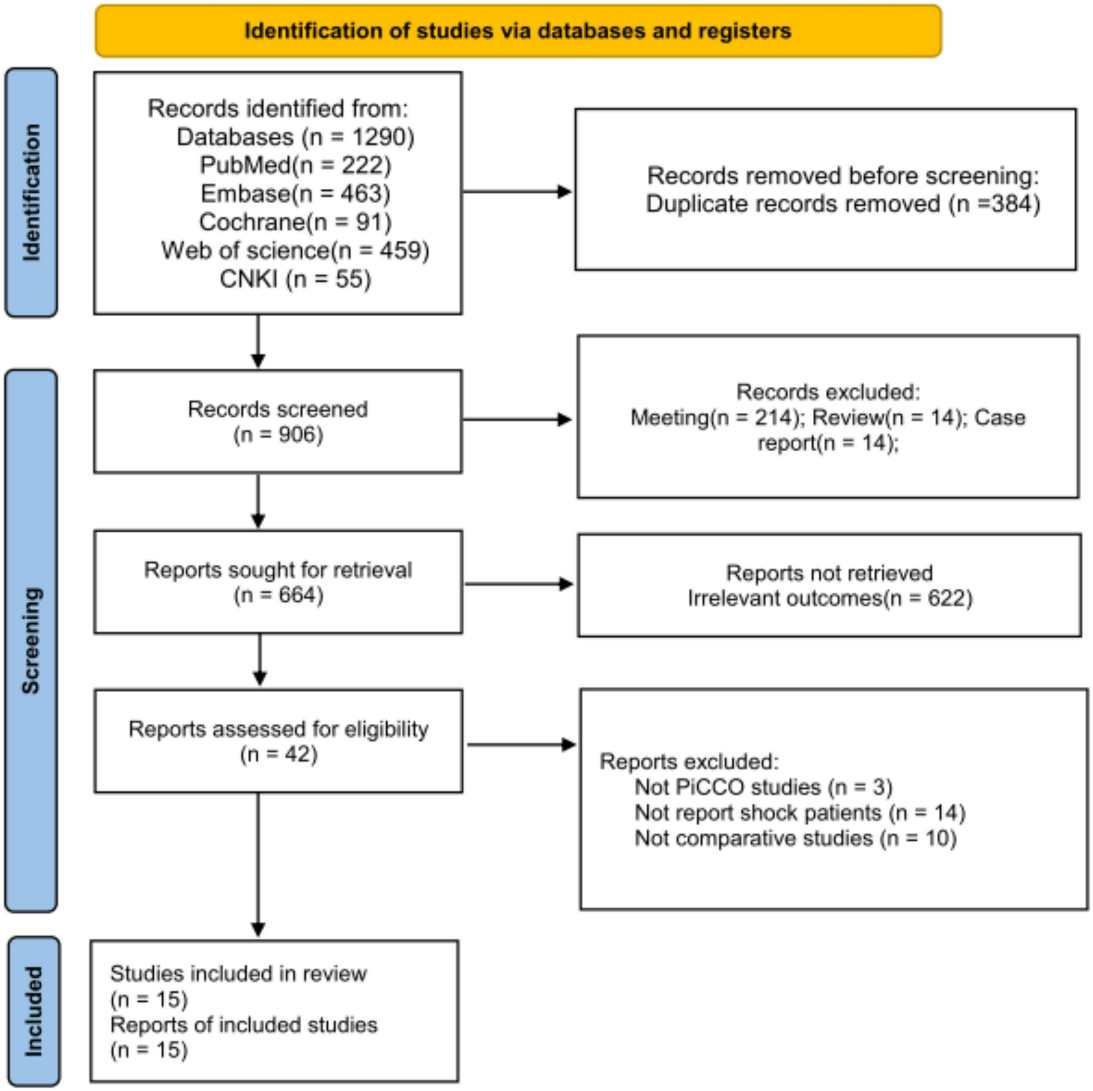
Flow diagram of the literature retrieve and selection.

The current study identified a total of 1,290 results, of which 15 studies published between 2013 and 2023 met the inclusion criteria (Figure 1) (8, 11–27). Among these, 7 were RCTs, 1 was prospective cohort study, and 7 were retrospective cohort studies. A total of 1,188 patients were enrolled in the meta-analysis, including 597 individuals that monitored by PICCO, which provides continuous CO monitoring, and 591 individuals by routine CVP monitoring, a traditional method that does not directly measure CO. However, none of the studies specified the exact duration of PiCCO catheter insertion or monitoring time. The patients included 747 male patients and 441 female patients with an average age from 32.0 to 70.6 years. The outcomes measures including length of ICU stay, duration of mechanical ventilation, 28-day mortality and other indexes were analyzed (Table 1). The risk of bias was judged as low for all studies, although several studies displayed high risk of selection bias due to random sequence generation and performance bias due to blinding of participants and personnel (Supplementary Figure S1).

**Table 1:** Characteristics of the included studies.

Author year	Country	Study design	Shock type	Patients number		Gender (M/F)		Mean Age (Years)		APACHE II		Outcome measures
				PICCO	Control	PICCO	Control	PICCO	Control	PICCO	Control	
Yu et al. (2021) (24)	China	Retrospective cohort	Traumatic shock	15	18	13/2	14/4	43	39	NR	NR	Fluid resuscitation volume; Lac; BE; oxygen index
Lin (2018) (12)	China	RCT	Traumatic shock	38	38	19/19	20/18	42.2	42.3	19.1	18.7	Cardiac index; HR; APACHE II; MAP
Yang (2021) (22)	China	RCT	Traumatic shock	43	43	22/21	20/23	47.1	46.4	NR	NR	Lac; Cardiac index; EVLW
Zhang (2021) (25)	China	Retrospective cohort	Traumatic shock	111	105	65/46	63/42	48.5	49	26.5	26.6	1*; 2*; 3*; APACHE II; fluid resuscitation volume; CVP; MAP; LAC
Lin (2021) (14)	China	RCT	Traumatic shock	50	50	27/23	28/22	48.7	48.9	NR	NR	1*; 2*; MAP; CVP; HR
Xue et al. (2013) (21)	China	Retrospective cohort	Traumatic shock	21	21	14/7	16/5	45.7	46.8	31.1	30.2	2*; APACHE II; MAP; LAC
Fang and Tang (2016) (11)	China	Retrospective cohort	Traumatic shock	18	18	13/5	12/6	43.87	45.32	20.8	24.7	2*; APACHE II; MAP; LAC
Shi et al. (2017) (20)	China	RCT	Traumatic shock	50	51	41/9	40/11	34	32	12	11	Mortality; APACHE II; fluid resuscitation volume; CVP; MAP; LAC
Meng et al. (2019) (18)	China	RCT	Traumatic shock	40	40	24/16	23/17	70.6	69.9	NR	NR	1*; fluid resuscitation volume; HR; CVP; MAP; LAC
Lin (2018) (13)	China	Retrospective cohort	Traumatic shock	30	30	18/12	17/13	45.28	45.59	NR	NR	MAP; Lactate clearance rate
Bian et al. (2021) (27)	China	Retrospective cohort	Traumatic shock	42	42	28/14	27/15	60.24	60.13	NR	NR	1*; fluid resuscitation volume; HR; CVP; MAP; LAC
Zhu et al. (2017) (40)	China	Retrospective cohort	Traumatic shock	45	41	29/16	31/10	41.8	43.1	16.5	17.1	1*; fluid resuscitation volume; HR; CVP; MAP; LAC; BE
Ma (2020) (17)	China	RCT	Traumatic shock	35	35	20/15	22/13	46.74	46.88	NR	NR	MAP; Lactate clearance rate
Ni (2022)	China	Prospective cohort	Traumatic shock	39	39	27/12	25/14	49.29	48.27	24.39	25.37	1*; 2*; 3*APACHE II; CVP; MAP
Yuan (2015)	China	RCT	Traumatic shock	20	20	14/6	15/5	60.7	59.2	21.65	21.8	1*; 2*; APACHE II; fluid resuscitation volume; CVP; MAP; LAC; HR

1*: length of ICU stay; 2*: duration of mechanical ventilation; 3*: 28-day mortality; 4: fluid resuscitation volume; RCT, randomized controlled trial; NR, not reported, missing values are indicated as “NR”; PICCO, pulse indicator continuous cardiac output; APACHE, Acute Physiology and Chronic Health Evaluation; SOFA, Sequential Organ Failure Assessment; CVP, central venous pressure; MAP, mean arterial pressure; BE, XXXX; LAC, lactate; HR, heart rates.

### The impact of PICCO monitoring on mortality of traumatic shock patients

**Figure 2:**
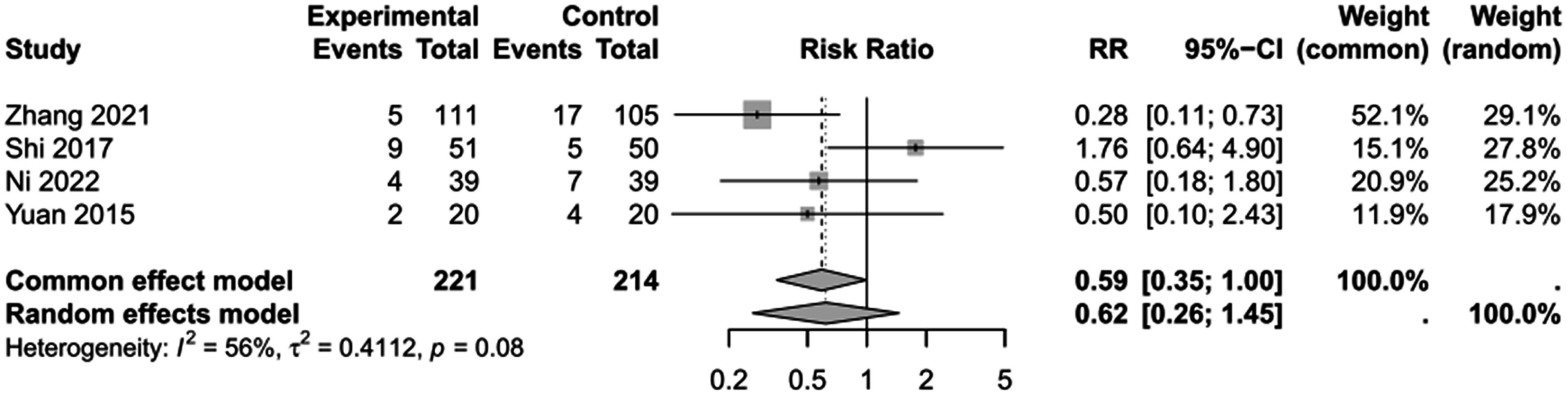
Forest plot: comparison of mortality.

**Figure 3:**
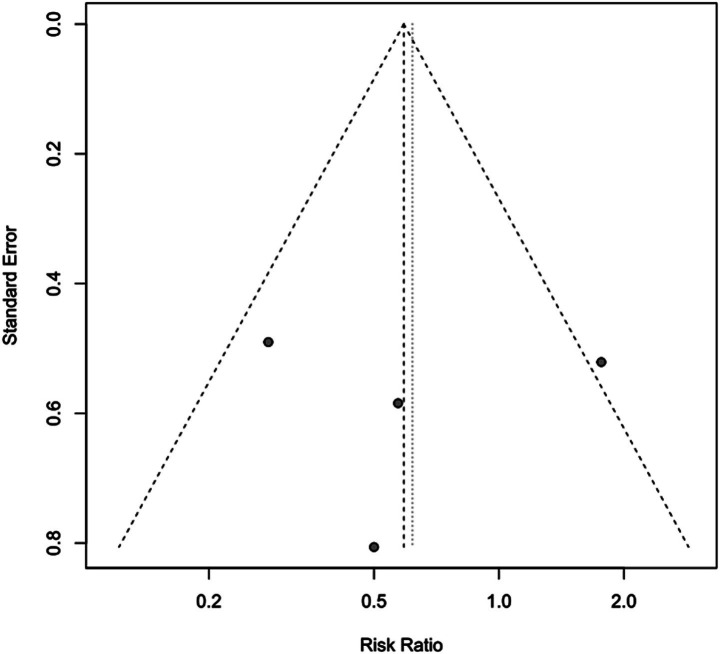
Funnel plot of mortality.

**Figure 4:**
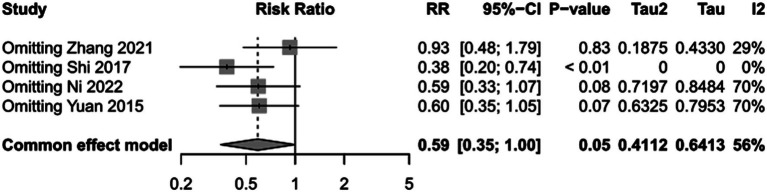
Sensitivity analysis of mortality.

To assess the impact of PICCO monitoring on mortality of traumatic shock patients, 4 studies (9, 19, 25, 28) comprising 221 cases in PICCO group and 214 cases in control group were analyzed, including 2 RCTs, 1 prospective cohort study and 1 retrospective cohort study. As shown in the forest plot in Figure 2, although the risk of mortality in PICCO group was lower when compared with the control group, the difference between the two groups was not significant [RR in random effects model: 0.62; 95% CI: (0.26, 1.45)]. The *I*^2^ = 56% indicated moderate heterogeneity in the meta-analysis. The funnel plot was created to identify the publication bias. The symmetry of the plot indicated the meta-analysis did not exhibit significant publication bias (Figure 3). The result of the influence analysis demonstrated the robustness of the analysis (Figure 4).

### The impact of PICCO monitoring on duration of mechanical ventilation of traumatic shock patients

**Figure 5:**
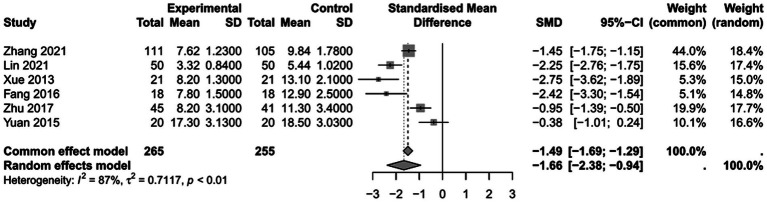
Forest plot: comparison of duration of mechanical ventilation.

**Figure 6:**
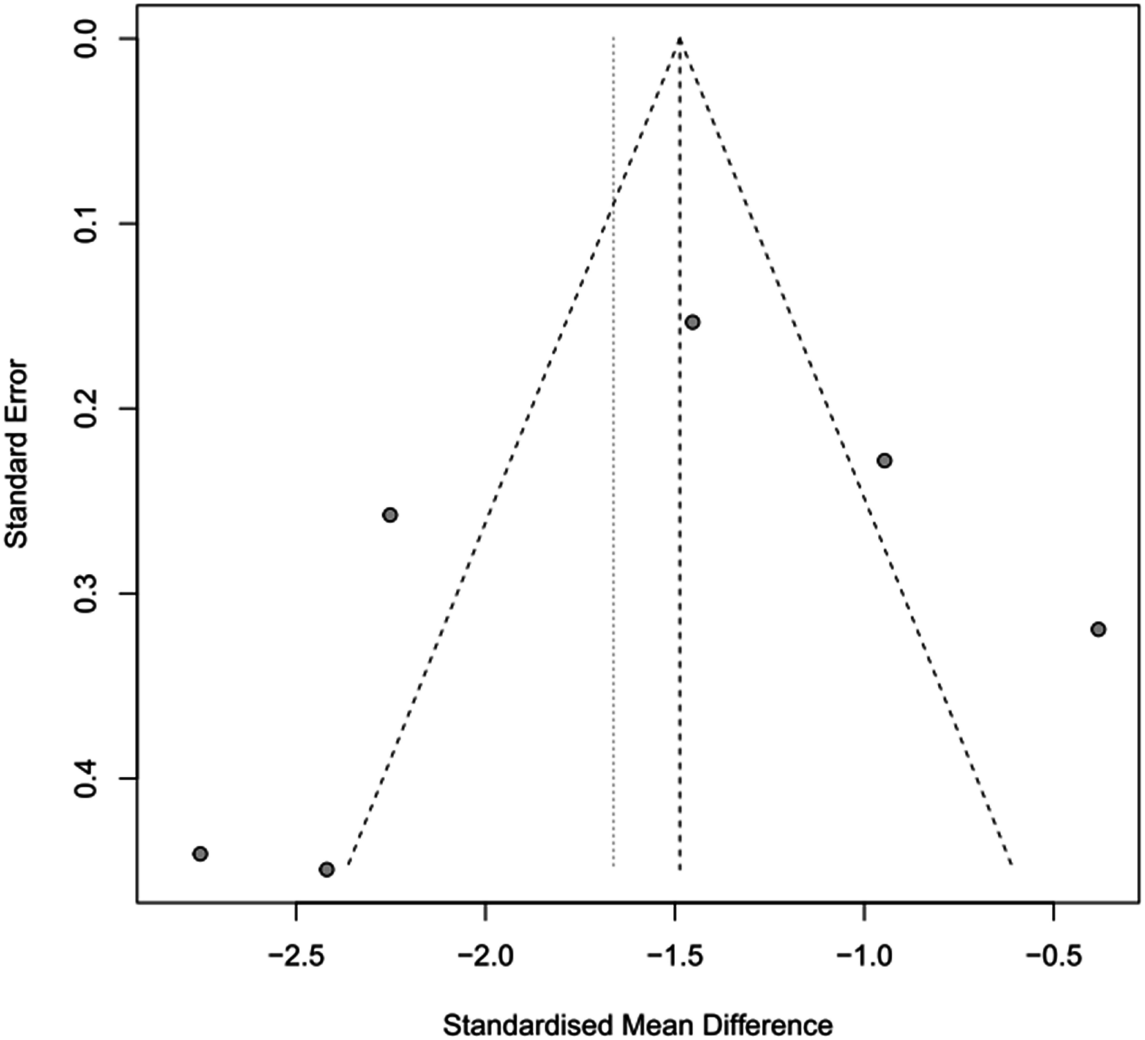
Funnel plot of duration of mechanical ventilation.

**Figure 7:**
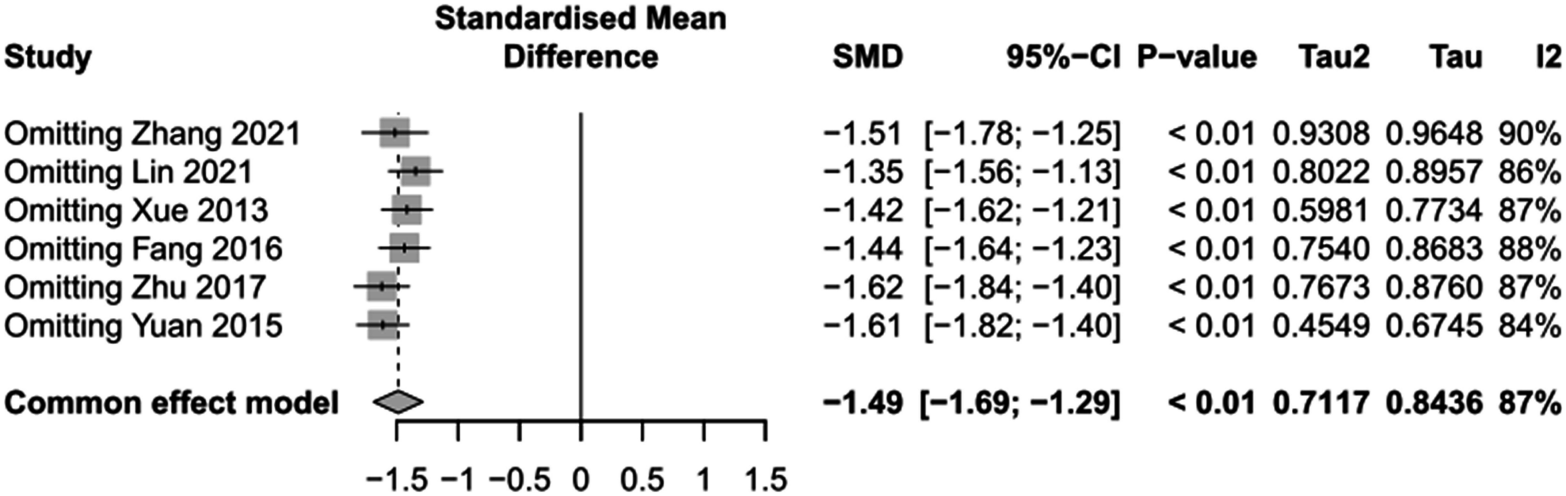
Sensitivity analysis of duration of mechanical ventilation.

To assess the impact of PICCO monitoring on duration of mechanical ventilation of traumatic shock patients, 6 studies (9, 19, 25, 26, 29, 30) comprising 265 cases in PICCO group and 255 cases in control group were analyzed, including 2 RCTs and 4 retrospective cohort studies. The forest plot in Figure 5 showed that the duration of mechanical ventilation in PICCO group was significantly lower than that in control group [SMD in random effects model: −1.66; 95% CI: (−2.38, −0.94)]. The *I*^2^ = 87% indicated obvious heterogeneity in the meta-analysis. The symmetry of the funnel plot indicated no obvious publication bias in the meta-analysis (Figure 6). Finally, the robustness of the analysis was examined by sensitivity analysis, and exclusion of any study at one time did not materially alter the overall estimates (Figure 7).

### The impact of PICCO monitoring on length of ICU stay of traumatic shock patients

**Figure 8:**
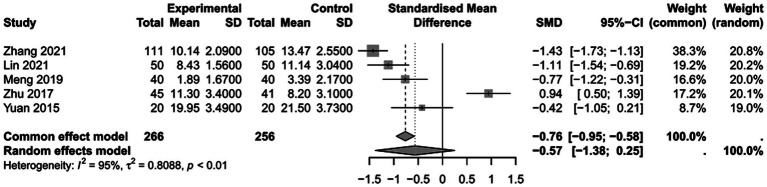
Forest plot: comparison of length of ICU stay.

**Figure 9:**
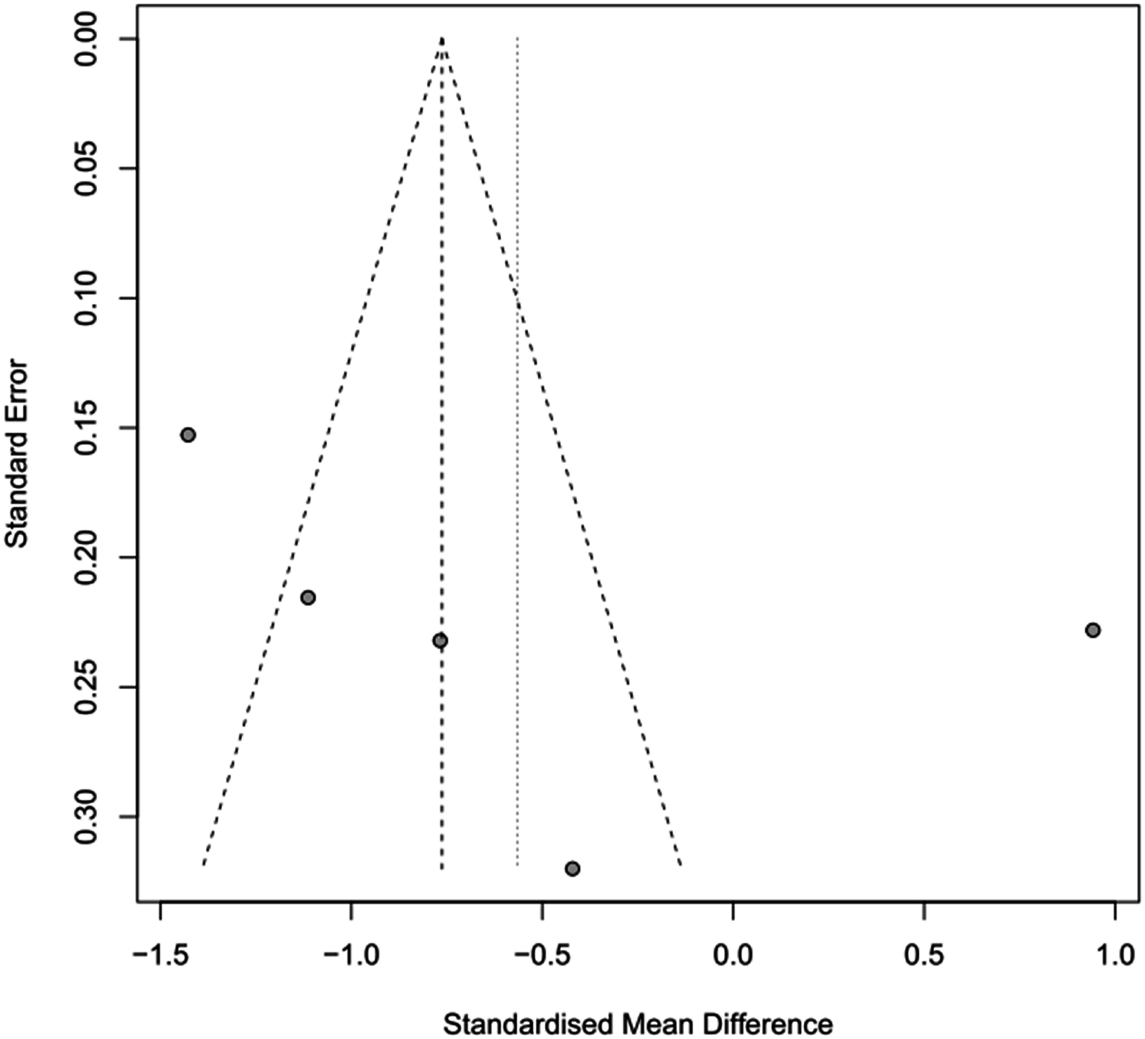
Funnel plot of duration of length of ICU stay.

**Figure 10:**
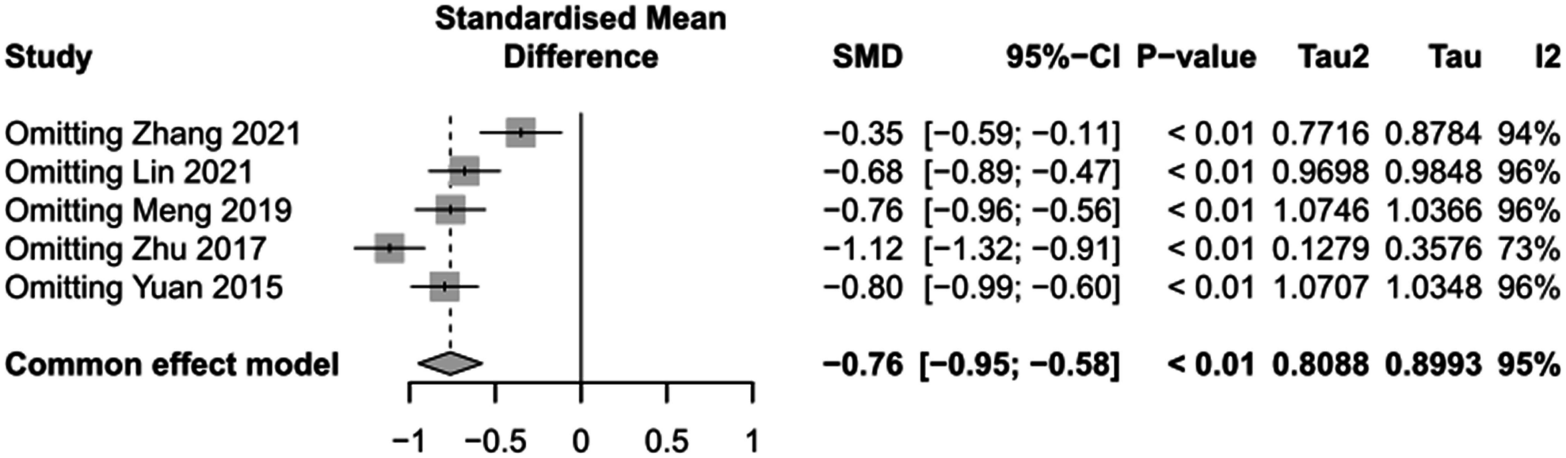
Sensitivity analysis of length of ICU stay.

To assess the the impact of PICCO monitoring on length of ICU stay of traumatic shock patients, 5 studies (9, 25, 26, 29, 31)comprising 266 cases in PICCO group and 256 cases in control group were analyzed, including 3 RCTs and 2 retrospective cohort studies. As shown in the forest plot, treatment based on PICCO displayed beneficial effect when compared with the control group, but the difference between the two groups was not significant [SMD in random effects model: −0.57; 95% CI: (−1.38, 0.25)]. To enhance interpretability, the SMD value was translated into clinical units: based on the pooled standard deviation of ICU stay (approximately 2.3 days across studies), this corresponds to an average reduction of about 1.3 days in ICU stay for the PICCO group compared to the control group. The *I*^2^ = 95% indicated significant heterogeneity in the meta-analysis (Figure 8). The funnel plot in Figure 9 demonstrated a moderate publication bias in the meta-analysis. The sensitivity analysis further demonstrated the robustness of the meta-analysis results, confirming that the overall conclusions were not significantly affected by the exclusion of individual studies (Figure 10).

### Meta regression

Meta-regression analyses were conducted for these three outcomes. Based on the availability of relevant data across included studies, meta-regression for 28-day mortality and duration of mechanical ventilation was performed according to study design and APACHE II stratification, while the analysis for length of ICU stay was conducted based solely on study design. The results indicated that neither study design nor APACHE II stratification accounted for the observed heterogeneity in any of the outcomes, suggesting that these factors were not significant sources of heterogeneity in our meta-analysis (Supplementary Figures S2–S4).

## Discussion

In this systematic review, a total of 1,290 studies were identified and 15 studies were included in the analysis. Based on these 15 studies, this systematic review revealed that traumatic shock patients monitored with PICCO exhibited lower mortality, reduced duration of mechanical ventilation, and shorter ICU stay. However, although a significant shorter duration of mechanical ventilation was observed, the difference between PICCO group and control group (CVP) in mortality and length of ICU stay was not statistically significant, suggesting that PICCO monitoring may improve the prognosis of traumatic shock to a certain extent. This study increases the understanding of the therapeutic effects of PICCO monitoring on traumatic shock patients through meta-analyzing the data of mortality, duration of mechanical ventilation, length of ICU stay. To our knowledge, this study represents the largest systematic review and meta-analysis of published studies of PICCO monitoring on traumatic shock population.

PICCO is one of the five most popular systems for arterial pulse contour analysis. The other four systems include FloTrac/Vigileo(®), LiDCO/PulseCO(®), PRAM/MostCare(®), and Modelflow (32–35). Several previous systematic reviews have demonstrated the benefits of PICCO monitoring, particularly in septic shock patients. For example, a meta-analysis in 2022 reported that PICCO monitoring can improve the prognosis of septic shock patients by shortening the duration of mechanical ventilation and ICU stay, and reducing 28-day mortality (36). Another 2014 study compared different arterial pulse contour analysis systems and found that PICCO showed acceptable accuracy during hemodynamically stable conditions (37). More recently, a 2023 meta-analysis demonstrated that PICCO monitoring in septic shock patients with ARDS resulted in improved oxygenation, shorter ICU stays and mechanical ventilation duration, fewer complications, and lower mortality rates (38). Additional evidence from a 2023 meta-analysis recommended clinical use of PICCO based on reduced use of vasopressors in purulent septic shock patients (39), and an earlier meta-analysis supported that PICCO monitoring was associated with shorter ICU stays and lower ICU and 28-day mortality in septic shock patients (40). However, in our current meta-analysis focusing on traumatic shock patients, only the duration of mechanical ventilation was significantly reduced in the PICCO group compared to the control group, while differences in mortality and ICU stay between groups were not statistically significant. This suggests that while PICCO monitoring may improve certain aspects of critical care, such as ventilatory support, its impact on overall survival and ICU length of stay in traumatic shock patients remains uncertain and may differ from its effects in septic shock.

Although based on these reported results, clinicians may be aware of the use of PICCO to improve prognosis of shock patients in ICU, the current study intend to explore the efficacy of PICCO in treatment of traumatic shock population. Studies have revealed that the intricate hemochemical makeup and changes during these shock states is exemplified in shock whether induced by infection or hemorrhage (41). PICCO can overcome the drawbacks of traditional CVP monitoring by continuously monitoring haemodynamics and providing a more comprehensive relevant parameters to guide the entire process of fluid resuscitation. PICCO’s impact on reducing the duration of mechanical ventilation may reflect better hemodynamic optimization, which potentially minimizes ventilator-associated complications by ensuring more precise fluid management and improved organ perfusion (42). This reduction in ventilation duration may be attributed to several mechanisms. First, PICCO allows for improved preload optimization by accurately assessing cardiac filling and guiding individualized fluid resuscitation, which helps avoid both hypovolemia and fluid overload (43). By preventing excessive fluid administration, PICCO can reduce the risk of pulmonary edema--a major contributor to prolonged mechanical ventilation in critically ill patients (44). Additionally, optimized fluid management via PICCO can enhance oxygen delivery to tissues, promoting faster recovery of organ function and reducing the need for prolonged ventilatory support (9). Collectively, these effects contribute to a more favorable respiratory profile, facilitating earlier liberation from mechanical ventilation. This aligns with the concept of protective hemodynamics, where continuous and comprehensive monitoring with PiCCO enables tailored interventions that support lung function and reduce the risk of ventilator-induced lung injury, thereby enhancing patient outcomes (45).

There are several possible explanations for why PICCO monitoring did not result in statistically significant reductions in mortality or ICU length of stay in our analysis. First, the presence of confounding variables--such as differences in baseline patient characteristics, variations in standard care across centers, and the use of concomitant therapies may have diluted the observable impact of PICCO on these outcomes. Second, the timing of intervention may play a crucial role; if PICCO monitoring is not initiated early enough in the course of shock, its potential benefits in preventing irreversible organ dysfunction or death may be limited. Third, many included studies enrolled patients with a wide range of illness severity, and PICCO’s advantages may be most pronounced in specific subgroups (e.g., those with more severe or refractory shock), which could have attenuated the overall effect in a heterogeneous population. Finally, mortality and ICU stay are influenced by numerous factors beyond hemodynamic monitoring, such as underlying comorbidities, complications, and institutional protocols, making it challenging to isolate the effect of PICCO alone on these endpoints.

Despite these promising findings, the study has several important limitations. First, all included studies were conducted in China, which limits generalizability due to global differences in trauma systems, ICU protocols, staffing, and resource availability. Second, key details regarding PICCO implementation—such as catheter duration and monitoring frequency—were frequently missing, limiting interpretation and reproducibility. Third, several studies lacked blinding, introducing potential performance and detection bias. Fourth, as most studies were observational, the risk of confounding and observational fallacy remains, since causality cannot be definitively established (46). Additionally, substantial heterogeneity was present among studies due to differences in shock definitions, patient selection, and study design. Finally, the small number of included studies makes it difficult to reliably assess publication bias. Collectively, these limitations underscore the need for more high-quality, multicenter randomized controlled trials (RCTs) in diverse healthcare settings to clarify the efficacy of PICCO monitoring in traumatic shock patients.

In conclusion, this systematic review and meta-analysis found that PICCO monitoring was associated with a significantly shorter duration of mechanical ventilation in patients with traumatic shock. However, no statistically significant difference was observed in mortality or ICU length of stay between the PICCO and control groups. While there appeared to be a trend toward improved prognosis with PICCO, further high-quality studies with larger sample sizes are needed to clarify its impact on mortality and other key clinical outcomes in this patient population. This meta-analysis provides preliminary evidence and highlights areas for future research regarding the use of PICCO monitoring in traumatic shock patients in the ICU.

## Data Availability

The original contributions presented in the study are included in the article/Supplementary material, further inquiries can be directed to the corresponding author.
