# Supplementary material for: The impact of PICCO monitoring on traumatic shock: a systematic review and meta-analysis
Source: Front Med (Lausanne). 2025 Nov 14;12:1578348. doi: 10.3389/fmed.2025.1578348 (PMC12660198; doi:10.3389/fmed.2025.1578348)
Supplement: Supplementary file 1 [file Data_Sheet_1.docx]

**Supplementary Table 1 (Table S1) Search strategy of each database.**

**Search strategy of PubMed**

| NO. | Search Details | Results |
| --- | --- | --- |
| #5 | #3 and #4 | 222 |
| #4 | "pulse indicator continuous cardiac output"[Title/Abstract] OR "PiCCO"[Title/Abstract] | 614 |
| #3 | #1 OR #2 | 1,844,426 |
| #2 | "shock hemorrhagic"[Title/Abstract] OR "hemorrhagic shock"[Title/Abstract] OR "shock"[Title/Abstract] OR "Hemorrhagic"[Title/Abstract] OR "trauma"[Title/Abstract] OR "injury"[Title/Abstract] | 1,354,948 |
| #1 | "traumatic shock"[Title/Abstract] OR "trauma induced shock"[Title/Abstract] OR "shock traumatic"[Title/Abstract] OR "traumatic"[Title/Abstract] OR ("Wounds"[Title/Abstract] AND "Injuries"[Title/Abstract]) OR "trauma*"[Title/Abstract] OR "injur*"[Title/Abstract] OR "wound*"[Title/Abstract] | 1,591,277 |

**Search strategy of EMBASE**

| No. | Query | Results |
| --- | --- | --- |
|  |  |  |
| #5 | #3 AND #4 | 463 |
| #4 | 'pulse indicator continuous cardiac output':ti,ab,kw OR 'picco':ti,ab,kw | 1288 |
| #3 | #1 OR #2 | 1937524 |
| #2 | 'hemorrhagic shock':ti,ab,kw OR 'shock':ti,ab,kw OR 'hemorrhagic':ti,ab,kw OR 'trauma':ti,ab,kw OR 'injury':ti,ab,kw | 1824415 |
| #1 | 'traumatic shock':ti,ab,kw OR 'trauma induced shock':ti,ab,kw OR 'shock traumatic':ti,ab,kw OR 'traumatic':ti,ab,kw OR 'wounds and injuries':ti,ab,kw | 289110 |

**Search strategy of Cochrane Controlled Register of Trials (CENTAL)**

| NO. | Search deatiles | Hits |
| --- | --- | --- |
| #1 | (traumatic shock):ti,ab,kw OR (trauma induced shock):ti,ab,kw OR (shock traumatic):ti,ab,kw OR (traumatic):ti,ab,kw OR (Wounds and Injuries):ti,ab,kw | 23595 |
| #2 | (Hemorrhagic shock):ti,ab,kw OR (shock):ti,ab,kw OR (hemorrhagic):ti,ab,kw OR (trauma):ti,ab,kw OR (injury):ti,ab,kw | 97242 |
| #3 | #1 OR #2 | 107036 |
| #4 | (pulse indicator continuous cardiac output):ti,ab,kw OR (PiCCO):ti,ab,kw | 178 |
| #5 | #3 and #4 in Trials | 91 |

Search strategy of web of science

| NO. | Search deatiles | Hits |
| --- | --- | --- |
| #1 | ((((TS=(traumatic shock)) OR TS=(trauma induced shock)) OR TS=(shock traumatic)) OR TS=(traumatic)) OR TS=(Wounds and Injuries) | 732991 |
| #2 | ((((TS=(Hemorrhagic shock)) OR TS=(shock)) OR TS=(hemorrhagic)) OR TS=(trauma)) OR TS=(injury) | 5789228 |
| #3 | #1 OR #2 | 5918064 |
| #4 | (TS=(pulse indicator continuous cardiac output)) OR TS=(PiCCO) | 1217 |
| #5 | #3 and #4 | 459 |

**Search strategy of CNKI**

(TKA=' PICCO' OR TKA=' PICCO' OR TKA=' pulse indicator continuous cardiac output') (TKA=' PICCO' OR TKA=' Pulse indicator continuous cardiac Output ') and (TKA=' traumatic shock' OR TKA='traumatic shock' OR TKA='shock traumatic' OR TKA='Hemorrhagic shock') 55

**Supplementary Figures**


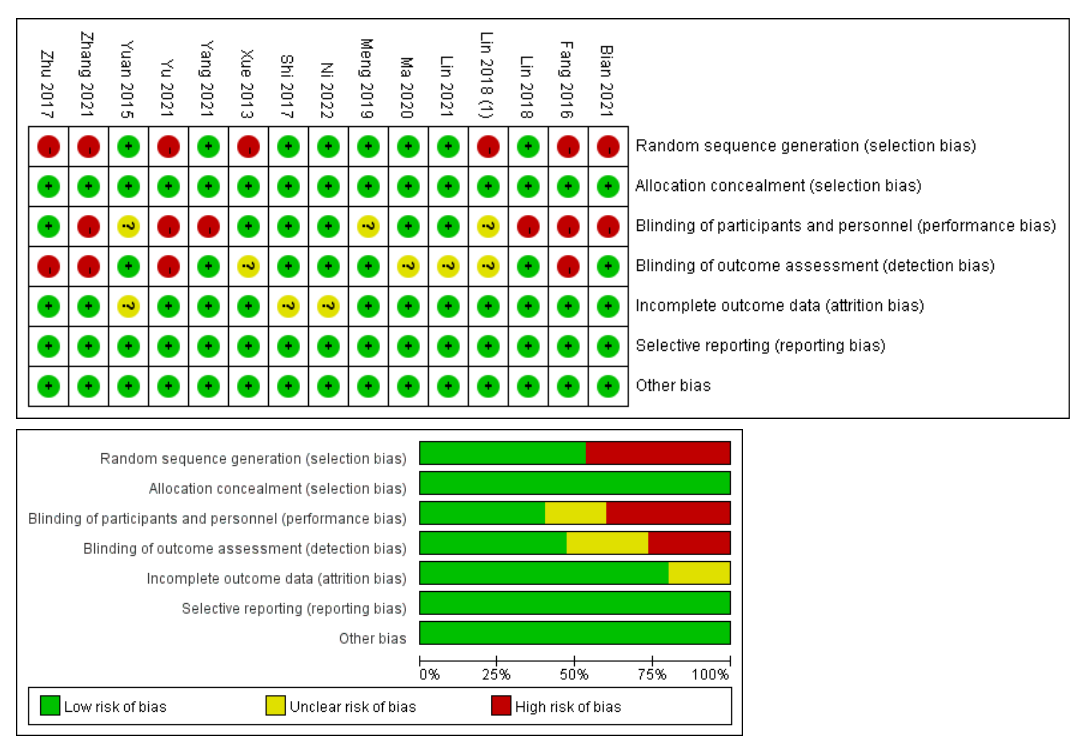


**Figure S1. Risk of bias of the included studies.**


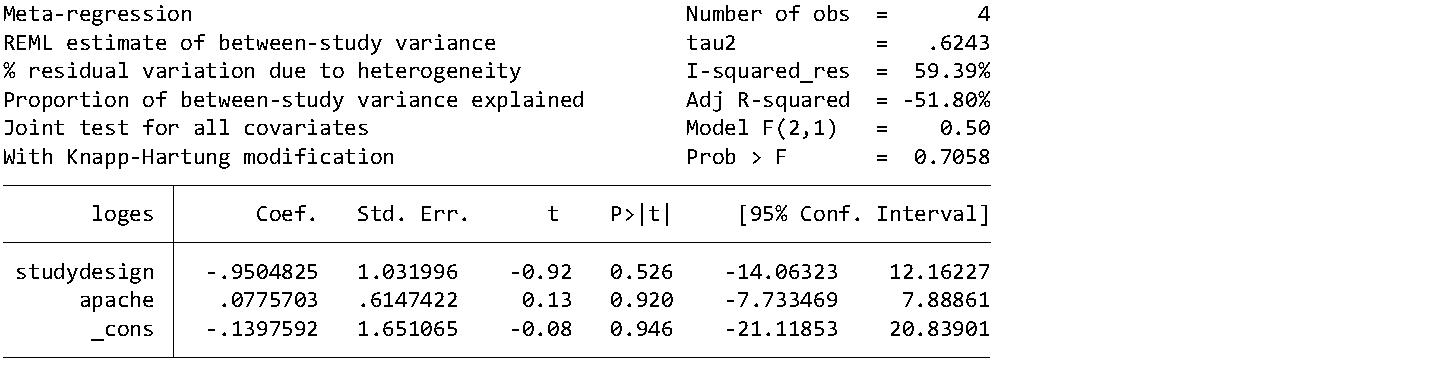


**Figure S2. Meta regression of mortality.**

**
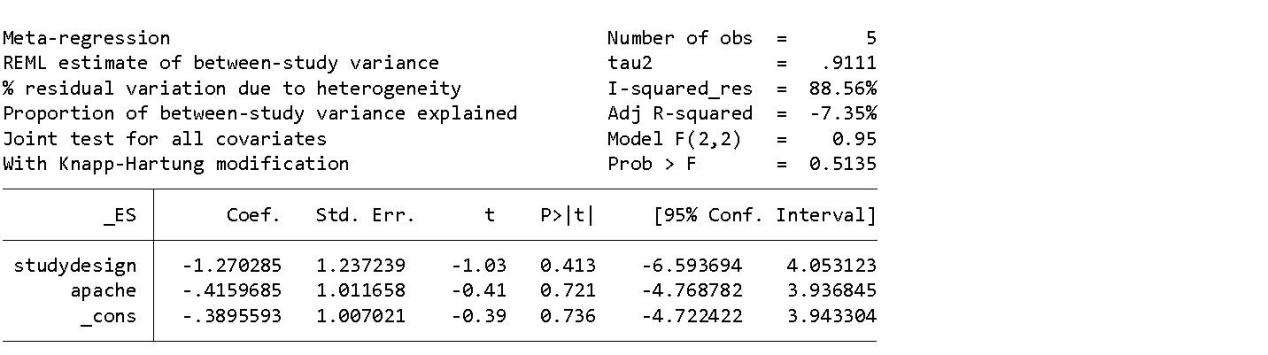
**

**Figure S3. Meta regression of mechanical ventilation.**

**
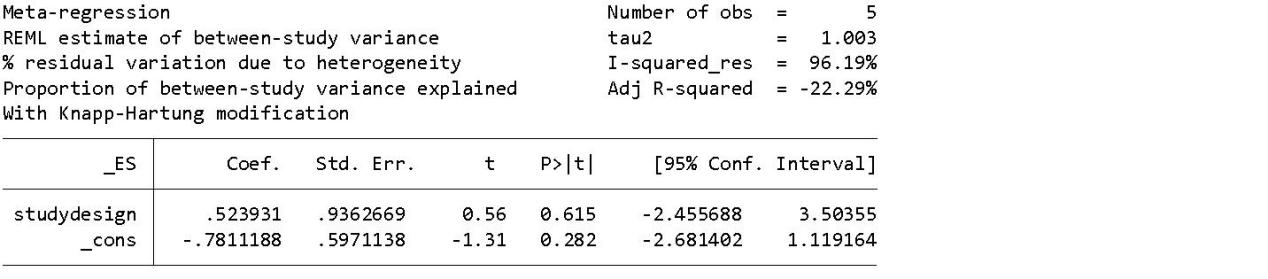
**

**Figure S4. Meta regression of ICU stay.**
